# Three-parent babies: Mitochondrial replacement therapies

**DOI:** 10.5935/1518-0557.20190086

**Published:** 2020

**Authors:** Hana Carolina Moreira Farnezi, Ana Carolina Xavier Goulart, Adriana dos Santos, Mariana Gontijo Ramos, Maria Lectícia Firpe Penna

**Affiliations:** 1 Faculdade de Ciências Humanas, Universidade FUMEC, Belo Horizonte, MG, Brazil

**Keywords:** mitochondrial, mtDNA, mitochondrial mutations, mitochondrial donation, mitochondrial replacement, reproductive technology

## Abstract

The mitochondria are intracellular organelles, and just like the cell nucleus they have their own genome. They are extremely important for normal body functioning and are responsible for ATP production - the main energy source for the cell. Mitochondrial diseases are associated with mutations in mitochondrial DNA and are inherited exclusively from the mother. They can affect organs that depend on energy metabolism, such as skeletal muscles, the cardiac system, the central nervous system, the endocrine system, the retina and liver, causing various incurable diseases. Mitochondrial replacement techniques provide women with mitochondrial defects a chance to have normal biological children. The goal of such treatment is to reconstruct functional oocytes and zygotes, in order to avoid the inheritance of mutated genes; for this the nuclear genome is withdrawn from an oocyte or zygotes, which carries mitochondrial mutations, and is implanted in a normal anucleated cell donor. Currently, the options of a couple to prevent the transmission of mitochondrial diseases are limited, and mitochondrial donation techniques provide women with mitochondrial defects a chance to have normal children. The nuclear genome can be transferred from oocytes or zygotes using techniques such as pronuclear transfer, spindle transfer, polar body transfer and germinal vesicle transfer. This study presents a review of developed mitochondrial substitution techniques, and its ability to prevent hereditary diseases.

## INTRODUCTION

The mitochondria are membrane-bound intracellular organelles present in almost all eukaryotic cells (Kasahara & Kato, 2018). They generate energy through oxidative phosphorylation, and are responsible for 90% of cellular ATP (Greenfield *et al*., 2017). In mammals, the mitochondria are present in all cells, except the enucleated red blood cells, being more present in tissues that need energy metabolism, with several units of the organelle. They have a round or oval shape and are about 0.5 to 1 µm in diameter, and up to 7 µm in length (Laureano, 2015). Together with the cell nucleus, they are the only cell organelle having their own genome, an extremely compact molecule, with 16.500 base pairs and 37 genes: 13 messenger RNAs, 22 RNAs, and 2 ribosomal RNAs. The D-loop is the only non-coding region in mtDNA, since introns and intergenic regions are non-existent or restricted to a few nucleotides (Chiaratti *et al*., 2018).

In addition to the production of reactive oxygen species due to the release of free electrons generated from the respiratory chain, mitochondria have few repair systems and therefore are subject to genetic mutations, causing diseases that affect approximately 1 in 5,000 people (Laureano, 2015). Mitochondrial diseases can affect organs that depend on energy metabolism, such as skeletal muscle, cardiac, central nervous system, endocrine, retina and liver (Nogueira *et al*., 2015; Laureano, 2015), giving rise to several incurable diseases, such as: deafness, diabetes mellitus, myopathies, glaucoma and others (Costa *et al*., 2016). These metabolic disorders, lead to inefficient oxidative phosphorylation, impairing cell energy production (Craven *et al*., 2017). They are difficult to diagnose and most of the time untreated, affecting adults and children (Nogueira *et al*., 2015).

Mitochondria are inherited only from the female gamete; therefore, the mitochondrial DNA is of exclusive maternal inheritance (Volobueva *et al*., 2018). The genetic mutations present in this material can be avoided using mitochondrial substitution techniques (Adashi & Cohen, 2018), where the nuclear genome is withdrawn from an oocyte, which carries mitochondrial mutations, and is implanted in a normal enucleated donor (Greenfield *et al*., 2017).

Mitochondrial donation techniques give women with mitochondrial defects a chance to have normal children (Greenfield *et al*., 2017). The proposed technique was approved in 2015 in the United Kingdom, with the first individual being born in Mexico. However, mismatches between mitochondrial and nuclear genomes during this process may occur (Eyre-Walker, 2017). The technique also faces theological and ethical dilemmas concerning the involvement of the donor with the generated embryo (Bianco & Montagna, 2016), since the child born will have three distinct genetic materials, one will come from the father through the spermatozoa, another from the biological mother, represented by nuclear DNA and the third by the donor of the cytoplasm containing mitochondrial DNA without pathological inheritance, generating a "three-parent baby".

Despite the benefits brought to the woman and family, the mitochondrial replacement technique is recent and not well discussed yet. Therefore, a better investigation is necessary. Thus, our study aims to present the mitochondrial substitution techniques to prevent hereditary diseases and discuss their possible outcomes.

## METHODS

**Figure 1:**
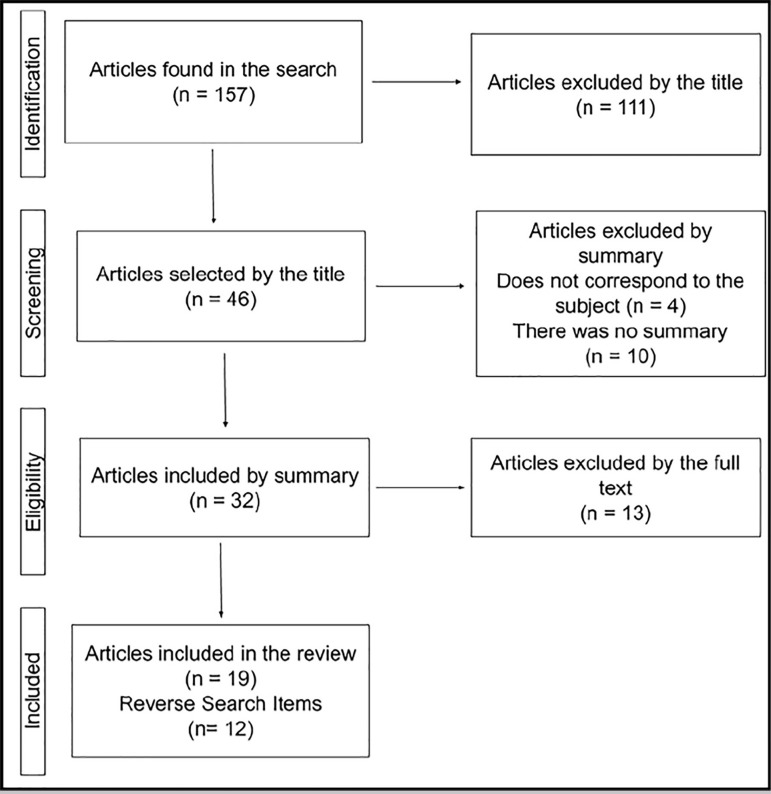
Flow chart representing the study’s methodology (Created by the author).

We performed an integrative review focused on the topic of mitochondrial substitution therapies. For this purpose we searched the PubMed database using the "mitochondrial DNA" and "replacement" terms. The search was restricted to research in humans in the last 10 years. The search yielded 157 results, from which we selected 46 abstracts. After reading all the abstracts, 33 papers were considered eligible. After reading the full texts, 19 papers were included in our review. Eleven other papers in the reverse search were considered in the construction of the paper (Fig. 1).

In the identification phase, we selected titles that corresponded to the researched topic, excluding those dealing only with diseases of mitochondrial inheritance. In the screening phase, those papers that did not have a summary available or that did not fit the theme were taken off. In the eligibility phase, the papers that answered some of the following predetermined questions about mitochondrial substitution techniques were considered:

1. Why study mitochondrial DNA?
2. Why perform the mitochondrial replacement therapy (MRT)?
3. What mitochondrial replacement techniques are known?
4. Is mitochondrial replacement therapy a good choice for the prevention of mitochondrial inheritance diseases?
5. Is mitochondrial replacement therapy a good option to improve the quality of eggs in women in advanced reproductive age?

## RESULTS

### 1. Why study mitochondrial DNA?

Diseases of mitochondrial origin, affecting 1 in 5,000 people in the world, have heterogeneous clinical manifestations and are difficult to diagnose and treat. The female gamete have significant amounts of mitochondria, 100,000 to 600,000 units, while male gametes have only 100, which are eliminated by an active process after fertilization, so the mother is the sole responsible for this heredity (Craven *et al*., 2017). 

Mitochondria can be all identical (homoplasmic) or different (heteroplasmic), having mutated and non-mutated material coexisting in the same cell. Thus, in women with homoplastic mutations in mtDNA, all offspring will be carriers of the mutation and all may develop symptoms; in women with heteroplasmic mutations in the mtDNA all their descendants could be affected, and the rate of heteroplasmy is important to determine the manifestation and severity of the disease (Craven *et al*., 2017). 

The severity of clinical symptoms is associated with the level of mtDNA mutation load (Zhang *et al*., 2017), and the most affected organs are those more dependent on energy metabolism, such as the brain, skeletal muscle, heart, and liver (Craven *et al*., 2011), so the risks of recurrence are difficult to estimate (Klopstock *et al*., 2016).

The first mutations in mitochondrial DNA (mtDNA) were demonstrated in 1988 and since then more than 200 mutations have been identified. They occur at a much higher rate than in nuclear DNA (nDNA), which can be due to three factors: generation of oxygen radicals by the respiratory chain, the absence of histones to protect DNA and few repair systems (Xu & Shi, 2016).

The heterogeneity of the manifestations and the amount of genes involved make diagnosis difficult, and the symptoms usually manifest when they have a 60 to 80% mutation rate; but those may vary (Craven *et al*., 2017). Finsterer *et al.* (2009) presented a guide for the molecular diagnosis of mitochondrial diseases. However, there is no cure for such disorders and palliative treatment with supplements, vitamins and coenzyme Q10, used to reduce symptoms, is of little value (Xu & Shi, 2016).

### 2. Why perform mitochondrial replacement therapy?

Currently, the options a couple has to prevent the transmission of diseases of mitochondrial origin are limited. These include adoption (of children or embryos), oocyte donation and induced abortion in specific cases of affected embryos after prenatal diagnosis (Zhang *et al*., 2017).

Pre-implantation genetic diagnosis (PGD) in assisted human reproduction (AHR) cases is considered as an option for the prevention of mitochondrial disorders. The test is made in one or more cells removed from an early embryo and enables embryo selection according to the mutation rate, before transfer to the uterus (Craven *et al*., 2017). However, PGD is not feasible for most cases, such as in women with homoplasmy (where all offspring will be affected) and high levels of heteroplasmy (due to the difficulty of selecting embryos with lower mutation load) (Mitalipov *et al*., 2014).

Treff *et al*. (2012) reported the case of PGD applied to a 30-year-old patient with a 35% mutation of mtDNA for mitochondrial encephalomyopathy with lactic acidosis and stroke-like episodes (MELAS), a 12% mutation embryo was transferred resulting at the birth of a boy. This boy presented several postpartum problems and samples collected at 6 weeks and 18 months showed heteroplasmic mutations for MELAS of 47% and 46% in blood and 52% and 42% in urine respectively (Mitalipov *et al*., 2014).

Cases such as this show that there are still uncertainties about the level of acceptable mtDNA heteroplasmy in the selection of embryos through PGD for the generation of healthy children. A phenomenon that contributes to the difficulty of identifying a safe percentage is the bottleneck effect (Mitalipov *et al*., 2014). The bottleneck is a hypothesis suggested from observations on changes in mtDNA heteroplasmy between generations and occurs in the ovary during oocyte development, possibly due to a marked reduction in the number of mtDNA copies in primordial germ cells, followed by segregation of mtDNA in mature oocytes (Wolf *et al*., 2017).

**Figure 2:**
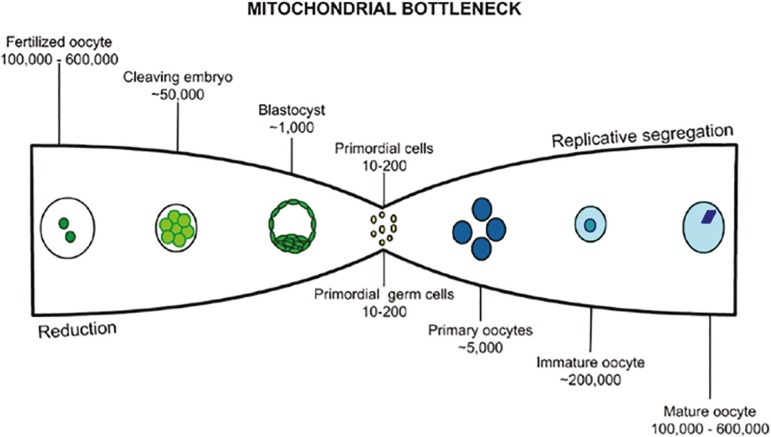
Bottle neck representation. The first bottle neck occurs in oocyte maturation, starting in primordial cells with 10 to 200 copies of mtDNA that replicate at random, to the mature oocyte with 100,000 to 600,000 copies. The second bottle neck occurs after oocyte fertilization by the spermatozoid, the zygote formed has 100,000 to 600,000 copies of mtDNA which are randomly reduced to the formation of primordial cells with 10 to 200 copies (Adapted from Craven *et al*., 2017).

The first bottleneck occurs during oocyte maturation in the ovary, where mature oocytes acquire different levels of mtDNA heteroplasmy. A new bottleneck occurs after fertilization of the oocyte, which may alter the level of heteroplasmy of the zygote that has 100,000 to 600,000 copies of mtDNA. There is a reduction in the mutations over the oocyte maturation, reaching approximately 50,000 at the cleavage stage, 1,000 at the blastocyst, and 10 to 200 copies at the primordial cell stage (Fig. 2) (Craven *et al*., 2017). The selection of the mtDNA that will be replicated is random, and further investigations are needed to identify the true nature of such phenomenon (Wolf *et al*., 2017). 

### 3. What techniques are known?

The technique of mitochondrial substitution, nuclear transfer (NT) or cytoplasmic transfer was initially proposed as a treatment for patients with infertility (Amato *et al*., 2014). Afterward, several techniques were developed in animal models to demonstrate different methods for mtDNA transfer and to check the viability of these techniques in reconstructing functional oocytes and zygotes, to avoid the inheritance of mutated genetic material (Craven *et al*., 2017).

Considering the results from these studies, the mitochondrial substitution technique may be an alternative not only for patients with infertility but also to enable women with pathogenic mutations in mtDNA to generate healthy offspring. For this, the nuclear genome is withdrawn from the oocyte or zygote carrying mutations in the mtDNA and transferred to a normal enucleated donor (Amato *et al*., 2014; Richardson *et al*., 2015; Wolf *et al*., 2015; Baylis, 2017; Craven *et al*., 2017).

The nuclear genome can be transferred from oocytes or zygotes using techniques such as pronuclear transfer (PNT), spindle transfer (ST), polar body transfer (PBT) and germinal vesicle transfer (GVT) (Craven *et al*., 2017; Wolf *et al*., 2015). The ooplasm transfer (OT) is also referred to as MRT (Yabuuchi *et al*., 2012; Cree & Loi, 2015; Craven *et al*., 2017), although not replacing the patient's mtDNA, only adding a small amount of mitochondria donor (Amato *et al*., 2014).

#### Pronuclear transfer

**Figure 3:**
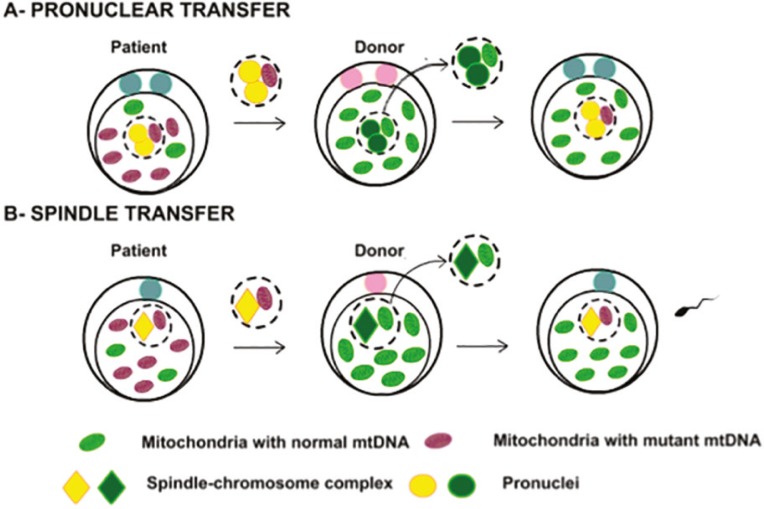
A - Representation of pronucleus transfer, where two PNs are withdrawn from the zygote with mutated mitochondria and transferred to an abnormal zygote that had its PNs previously removed. B - Representation of the maternal spindle transfer, where the spindle is removed and transferred to an oocyte in MII that had the spindle previously removed, followed by fertilization of the reconstructed oocyte (Adapted from Craven *et al*., 2017).

After the oocyte fertilization, two pronuclei (PN) with defined membranes containing each a haploid set of chromosomes become visible and migrate to the center of the zygote (Wolf *et al*., 2015; Craven *et al*., 2017). In the PNT, the two PN are removed from the zygote with mutated mitochondria and transferred to a normal zygote that had their PN previously removed (Fig. 3a) (Cree & Loi, 2015; Klopstock *et al*., 2016; Craven *et al*., 2017; Wolf *et al*., 2017). McGrath & Solter (1983) developed the first NT technique using rat zygotes, which has been performed to this day. The fusion between PN and cytoplasm is done by electrical pulse or inactivated Sendai virus (Yabuuchi *et al*., 2012; Amato *et al*., 2014; Cree & Loi, 2015).

To ensure nucleus integrity in PNT, it is inevitable that a small amount of mutated mitochondria is transferred, resulting in heteroplasmy (Xu & Shi, 2016; Wolf *et al*., 2017). The technique is related to a large transfer of residual mtDNA in rat models but low level in human embryo (Wolf *et al*., 2015). In the first human study using the technique, published by Craven *et al*. (2010), abnormal human zygotes (1 or 3 PN) were used, and this technique was able to reduce to 2% the rate of mutant mtDNA in most reconstructed zygotes.

Zhang *et al*. (2016) applied the PNT technique and obtained a total of 7 zygotes successfully rebuilt, 5 of which were transferred to the patient, resulting in a triple pregnancy. Although the pregnancy did not go to term, the fetuses did not present heteroplasmy and exams showed normal karyotype.

Hyslop *et al*. (2016) showed that performing PNT earlier, after meiosis completion, improves the zygote survival rate. The study also suggested that oocyte vitrification will minimize mtDNA transport in the procedure.

#### Spindle transfer

The maternal spindle, or spindle-chromosome metaphase II (MII) complex, is formed within oocytes during the second meiotic division (Craven *et al*., 2017). In the MST the spindle is removed and transferred to an oocyte in MII that had the spindle previously removed (Fig. 3b) (Tachibana *et al*., 2009; Cree & Loi, 2015; Wolf & Mitalipov, 2014; Kang *et al*., 2016; Wolf *et al*., 2017; Craven *et al*., 2017; Zhang *et al*., 2017). The fusion between the cytoplasm and the spindle is induced by electrofusion or inactivated Sendai virus (Cree & Loi, 2015).

The first study using ST in humans was published by Tachibana *et al*. (2012), and it confirmed the viability of the technique despite the high rate of abnormal fertilization in zygotes after oocyte manipulation (52%), since the percentage was similar to that of frozen controls.

Kang *et al*. (2016) were the first to use ST in human oocytes from women carrying a pathogenic mutation in mtDNA. The study showed a level of fertilization and blastulation similar to that of unmanipulated oocytes and the zygotes formed contained more than 99% of donated mtDNA, demonstrating the viability of the technique.

Zhang *et al*. (2017) performed MST in an asymptomatic Leigh syndrome woman with a history of two deceased children (with Leigh syndrome confirmed with more than 95% mutation in mtDNA) and four miscarriages. Five oocytes were successfully reconstructed and fertilized, four developed to blastocyst, but only one presented normal karyotype and was implanted, resulting in the birth of a boy with less than 6% of heteroplasia.

#### Polar body transfer

The polar body (PB) is a small residual structure of the first meiotic division. The first polar body (PBI) appears after ovulation, the second polar body (PBII) arises after fertilization with the spermatozoa, and both contain an exact genetic copy of the oocyte nucleus (Wang *et al*., 2014).

**Figure 4:**
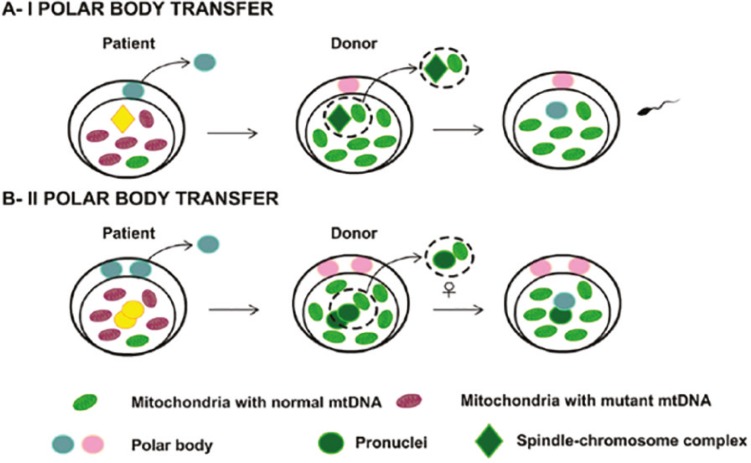
A - PBI transfer Representation, where it is withdrawn from the oocyte in MII and transferred to an oocyte in MII with the spindle previously removed, followed by fertilization of the reconstructed oocyte. B - Representation a PBII transfer, where it is removed from an azygote in the pronucleus stage and transferred to azygote with the maternal nucleus and PBII previously removed (Adapted from Craven *et al*., 2017).

In the PBI transfer (TPBI), the polar body was withdrawn from the oocyte in MII and transferred to an oocyte in MII with the spindle previously removed, followed by fertilization of the reconstructed oocyte (Fig. 4a). On PBII transfer (TPBII), the polar body is removed from a zygote in the pronucleus stage and transferred to a zygote with the maternal nucleus and the PBII previously removed (Fig. 4b). Due to the small amount of residual mtDNA transferred in PBT the technique has great potential for disease prevention (Wang *et al*., 2014).

Ma *et al.* (2017) produced functional oocytes using TPBI, five of them presenting chromosomal spindle, but only two of them showed a metaphase spindle similar to oocytes in intact MII. The reconstructed oocytes were fertilized by intracytoplasmic sperm injection (ICSI) and 76% of the zygotes obtained had two PN and the PBII, indicating normal fertilization.

There is no record of TPBII using human zygotes, probably due to the difficulty in differentiating the maternal and paternal nuclei, making it hard to select and correctly extract the maternal nucleus to complete the technique (Craven *et al*., 2017).

#### Germinal vesicle transfer

**Figure 5:**
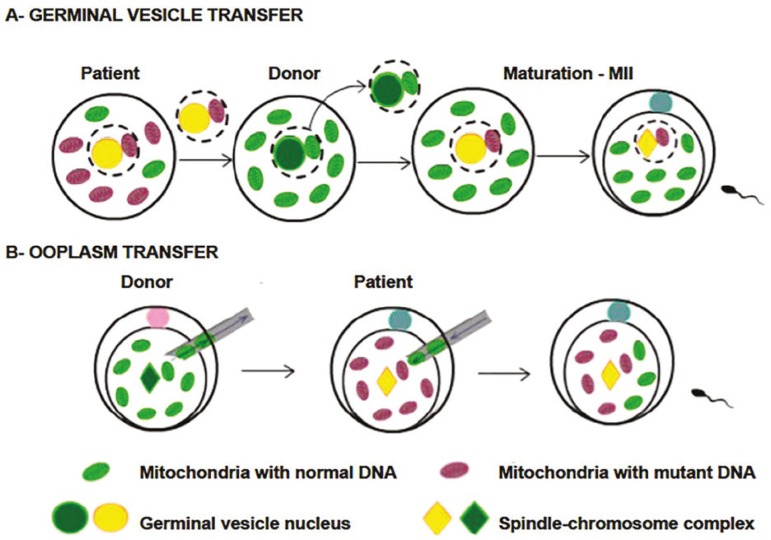
A - Terminal vesicle transfer representation, where the germinal vesicle I is removed and transferred to an oocyte that had its germinal vesicle previously removed, then the constructed oocyte undergoes *in vitro* maturation followed by fertilization. B - Plasma transfer representation, which consists of transferring from 5 to 15% of the cytoplasm content of a healthy donor to the oocyte of the patient with mutated mitochondria, followed by oocyte fertilization (Adapted from Craven *et al*. 2017).

The germinal vesicle is the nucleus of an immature oocyte in prophase I of the first meiosis. In the GVT the germinal vesicle is removed and transferred to an oocyte that had its germinal vesicle previously removed, and then the reconstructed oocyte undergoes in-vitro maturation followed by fertilization (Fig. 5a). The technique was initially developed to treat infertility in older women (Cree & Loi, 2015).

#### Ooplasm transfer

The OT or cytoplasmic transfer was the first technique created to treat infertility (Amato *et al*., 2014), and it consists of transferring from 5 to 15% of the cytoplasm content of a healthy donor, containing mtDNA, proteins, mitochondria and other organelles, to the oocyte of the patient with mutated mitochondria (Fig. 5b) (Yabuuchi *et al*., 2012; Cree & Loi, 2015; Amato *et al*., 2014; Craven *et al*., 2017). The technique was initially developed using animal models with the intention of improving oocytes with low cytoplasmic quality (Craven *et al*., 2017).

Despite controversies about OT, it has been used as an ART and has led to almost 50 pregnancies (Craven *et al*., 2017), and in the late 1990s it resulted in the birth of more than 30 babies (Cree & Loi, 2015).

### 4. Is mitochondrial replacement therapy a good choice for the prevention of mitochondrial inheritance diseases?

Due to the small amount of mutated mitochondria loaded during the execution of MRT, and the bottleneck effect, the technique may decrease the chance of disease transmission. However, it cannot secure its prevention, and as the mutation load may be different from cell to cell and from tissue to tissue, recurrence risks are difficult to estimate (Klopstock *et al*., 2016). Nonetheless, considering the success of MRT implementation (Craven *et al*., 2010; Zhang *et al*., 2016; 2017; Ma *et al*., 2017), there is no question that the technique has a large potential to be explored in preventing the transmission of mtDNA-related diseases.

Craven *et al*. (2010) showed that human zygotes can be reconstructed through PNT, with potential to prevent the transmission of diseases related to mtDNA. Zhang *et al*. (2016) also reported that the same technique resulted in viable pregnancy with normal karyotype and low heteroplasia rate.

Ma *et al*. (2017) suggested that MRT needs to undergo regulatory approval prior to application in clinics, and showed that, in conjunction with ST, PBIT can potentially increase the number of reconstructed oocytes for women with mtDNA defects.

For Zhang *et al*. (2017) there is certainly much controversy about MRT, and more studies are required. However, the first live birth reported by them strongly suggests that ST can significantly reduce the rate of mutated mtDNA in offspring.

Questions were raised about the communication between the mother's nDNA and the donor's mtDNA, and the potential deleterious effects of mixing genomes and potential incompatibilities between them. However, there is little evidence that MRT is detrimental due to deleterious mito-nuclear interactions, since the procedure in humans leads to normal gene expression in blastocysts. In addition, in normal reproduction, the interaction of the mitochondria with the nuclear environment is new, since they have never previously experienced half of the father's nuclear genome. Therefore, if this process occurs normally, it is possible that the interaction between half of the nDNA donated by the mother will happen in the same way (Eyre-Walker, 2017).

### 5. Is mitochondrial replacement therapy a good option to improve the quality of eggs in women of advanced reproductive age?

The advanced age of the woman is one of the main factors responsible for infertility, embryonic and fetal losses (Wolf *et al*., 2015). Their oocytes have few copies of mtDNA when compared to the number of copies found in younger women (Craven *et al*., 2017). In addition, they have high rates of aneuploidy, one of the main causes of female infertility (Cree & Loi, 2015). These factors decrease the efficacy of conventional assisted reproduction techniques that depend on oocyte quality and number (Ma *et al*., 2017).

Fragouli *et al*. (2015) reported that in embryos from older women, the levels of mtDNA are lower at the cleavage stage and higher at the blastocyst stage, when compared to those from younger women. A possible explanation for this increased proliferation is that it occurs as a compensation for mitochondrial dysfunction (Schon & DiMauro, 2003).

Given these relationships between mitochondria and infertility, the introduction of mitochondria donated by a younger woman may have the potential to restore the fertility of older woman's eggs. Cohen *et al*. (1997) were the first to report OT from human oocytes resulting in pregnancy in a 39-year-old patient with a history of 5-6 years of infertility. OT was performed on 14 oocytes, with material transferred from a 27-year-old donor, and 6 reconstructed oocytes showed normal development after fertilization, and transference of the zygotes resulted in the birth of a girl.

Although TPBI cannot be used in women who do not produce mature oocytes, the technique may improve the outcomes of assisted reproduction treatment and pregnancy rates, especially for older women with decreased ovarian reserve (Ma *et al*., 2017).

GVT was initially created with the intention of avoiding aneuploidy in oocyte-derived embryos of older women (Cree & Loi, 2015). Tanaka *et al*. (2009) reported the reconstruction of 35 oocytes of older women using enucleated oocytes from young women, 28% of these reconstructed oocytes developed for blastocyst, while only 3% showed this development in the untreated control group.

## CONCLUSION

Diseases caused by mtDNA may manifest in children and adults causing irreparable damage and, due to the lack of available treatments, prevention is the only option for the inheritance control. Due to the multiplication pattern of the mitochondria during the embryonic development, the PGD did not present reliable results concerning the prevention of mutation transmission, once the mtDNA rate for an embryo to develop without manifesting the disease is not known. Women with homoplastic mtDNA cannot benefit from PGD either, since all their offspring will be affected.

MRTs were initially developed for the treatment of infertility in older women and then improved for the prevention of mitochondrial diseases. Despite reports with positive results, there are still questions about the amount of mtDNA load during the procedure. Therefore more studies must be performed in humans to prove their effectiveness in preventing the transmission of mitochondrial disorders.
